# Development and validation of prediction models for death within 6 months after cardiac arrest

**DOI:** 10.3389/fcvm.2024.1469801

**Published:** 2024-11-28

**Authors:** Jianping Lu, Yuqi Zeng, Nan Lin, Qinyong Ye

**Affiliations:** ^1^ Department of Neurology, Fujian Medical University Union Hospital, Fuzhou, China; ^2^ Institute of Clinical Neurology, Fujian Medical University Union Hospital, Fuzhou, China; ^3^ Department of Geriatrics, Fujian Medical University Union Hospital, Fuzhou, China

**Keywords:** cardiac arrest, prognosis, prediction model, machine learning, return of spontaneous circulation

## Abstract

**Background:**

Even in patients with a successful return of spontaneous circulation (ROSC), outcomes after cardiac arrest (CA) remain poor, with some eventually succumbing after several months of treatment. There is a need for early assessment of outcomes in patients with ROSC after CA. Therefore, we developed three models for predicting death within 6 months after CA using early post-arrest factors, performed external validation, and compared their efficiency.

**Methods:**

In this retrospective cohort study, 199 patients aged 18–80 years who experienced either in-hospital cardiac arrest or out-of-hospital cardiac arrest and achieved ROSC were included as the training set. Patients were divided into an “alive” group (95 cases) and a “dead” group (104 cases) according to their survival status 6 months after CA. Demographic data, medical history, and laboratory results were collected. Univariate and multivariate logistic regression analyses were used to identify risk factors. A risk prediction model was constructed using random forest methods, support vector machine (SVM), and a nomogram based on factors with *P* < 0.1 in the multivariate logistic analyses. An additional 42 patients aged 18–80 years who experienced CA with ROSC were included as the validation set. Receiver operating characteristic (ROC), decision, and calibration curves were used to assess model performance.

**Results:**

Duration of cardiac arrest, lactate level after ROSC, secondary infections, length of hospital stay, and ventilator support were the top five risk factors for death within 6 months after CA (*P* < 0.1) in sequence. The random forest model [average area under the ROC curve (AUC), training set = 0.991, validation set = 0.703] performed better than the SVM model (AUC, training set = 0.905, validation set = 0.636) and the nomogram model (AUC, training set = 0.893, validation set = 0.682). Decision curve analysis indicated that the random forest model provided the best net benefit. The calibration curve indicated that the prediction for death within 6 months after CA by the random forest model was consistent with actual outcomes. The AUC of the prediction model constructed using random forest, SVM, and nomogram methods was 0.991, 0.893, and 0.905, respectively.

**Conclusions:**

The prediction model established by early post-arrest factors performed well, which can aid in evaluating prognosis within 6 months after cardiac arrest. The predictive model constructed using random forest methods exhibited better predictive efficacy.

## Introduction

Cardiac arrest (CA) is the sudden cessation of cardiac ejection function and carries low survival rates or unfavorable outcomes (1–3). It is a significant public health problem and the leading cause of death worldwide (4–6). A study reported that the annual incidence of CA in the United States is approximately 150,000 cases, claiming thousands of lives. Each year, approximately 292,000 adults suffer an in-hospital cardiac arrest (IHCA) (7), while 420,000 people suffer an out-of-hospital cardiac arrest (OHCA) (8). In another study including 24,132 patients admitted to critical care units after CA in the United Kingdom, the in-hospital mortality rate was 71% (9). In China, an estimated 54.4 million people experience CA each year, of which only about 1% of people survive (10). Even worse, the global incidence of CA continues to increase each year (11, 12).

A large number of CA patients with return of spontaneous circulation (ROSC) undergo continuous monitoring and relevant treatment in the intensive care unit (ICU) (13–16). Therefore, clinicians rely on medical history and various examination results to make early prognostic assessments and provide adequate counseling to relatives. By far, several prognostic models have been used to predict prognosis for CA patients (17–19). Even in patients with successful ROSC, outcomes after CA remain poor, with some patients ultimately succumbing after several months of treatment (20, 21). Although substantial progress has been made in the prognostication of short-term outcomes after cardiac arrest, early post-arrest factors predicting death within 6 months after CA are still unreported. There is a need for an early assessment of outcomes in patients with ROSC after CA.

Therefore, this study aimed to develop models for predicting death within 6 months after CA using early post-arrest factors, perform external validation, and compare their efficiency.

## Methods

### Study population

This retrospective cohort study included 264 patients aged 18–80 years who experienced either IHCA or OHCA and achieved ROSC at Fujian Medical University Union Hospital. Patients admitted from January 2018 to June 2022 were used as the training set, while those admitted from July 2022 to December 2023 were used as the validation set. Informed consent was not required because this study involved a retrospective review of medical records. The study was approved by the Ethics Committee of Fujian Medical University Union Hospital (2021-KJT045).

CA was identified using ICD-10 codes corresponding to cardiac arrest or resuscitation, such as I46.0 (cardiac arrest with successful resuscitation), I46.1 (sudden cardiac death), and I46.9 (cardiac arrest, unspecified). Patients with ICD codes for cardiac arrest or resuscitation who had invasive rescue efforts refused by relatives, patients without initially recorded vital signs, and patients who arrived at the emergency department (ED) with unstable initial vital signs indicating peri-arrest status (systolic blood pressure ≤ 40 mmHg; heart rate ≤ 20/min; and respiratory rate ≤ 4/min) were all excluded. In addition, patients younger than 18 years of age or older than 80 years were excluded. In this study, secondary infection refers to an infection that occurred during the first hospitalization after cardiac arrest. The interval from the beginning of cardiac arrest to the return of spontaneous circulation was defined as the duration of cardiac arrest.

### Data collection

Blood samples were collected within 24 h after CA, and lactate levels were measured. All clinical data were collected from clinical electronic medical records and extracted into Microsoft Excel for later analysis. Data regarding demographic and clinical characteristics were collected. Follow-up phone interviews were conducted 6 months after CA. The main outcome was death, either in-hospital or out-of-hospital, within 6 months after CA.

### Statistical analysis

#### Identify risk factors

Continuous variables were presented as mean ± standard deviation, and categorical variables were presented as frequency (%). We compared baseline characteristics between the alive and dead groups using the Wilcoxon two-sample test, chi-square test, or Fisher's exact test. Multivariate logistic regression was performed using IBM SPSS Statistics 26 to identify risk factors.

#### Model development and external validation

Factors with *P* < 0.1 in multivariate analysis were used to establish the predictive model using the rms package in R4.3.1 software. Receiver operating characteristic (ROC) curves for each factor and the multifactor model were plotted, and the area under the ROC curve (AUC) values were calculated. The calibration curve and the nomogram were plotted using the “rms” package, and the decision curve was generated using the “rmda” package in R4.3.1 software.

## Results

### Risk factors for death within 6 months after CA

**Figure 1:**
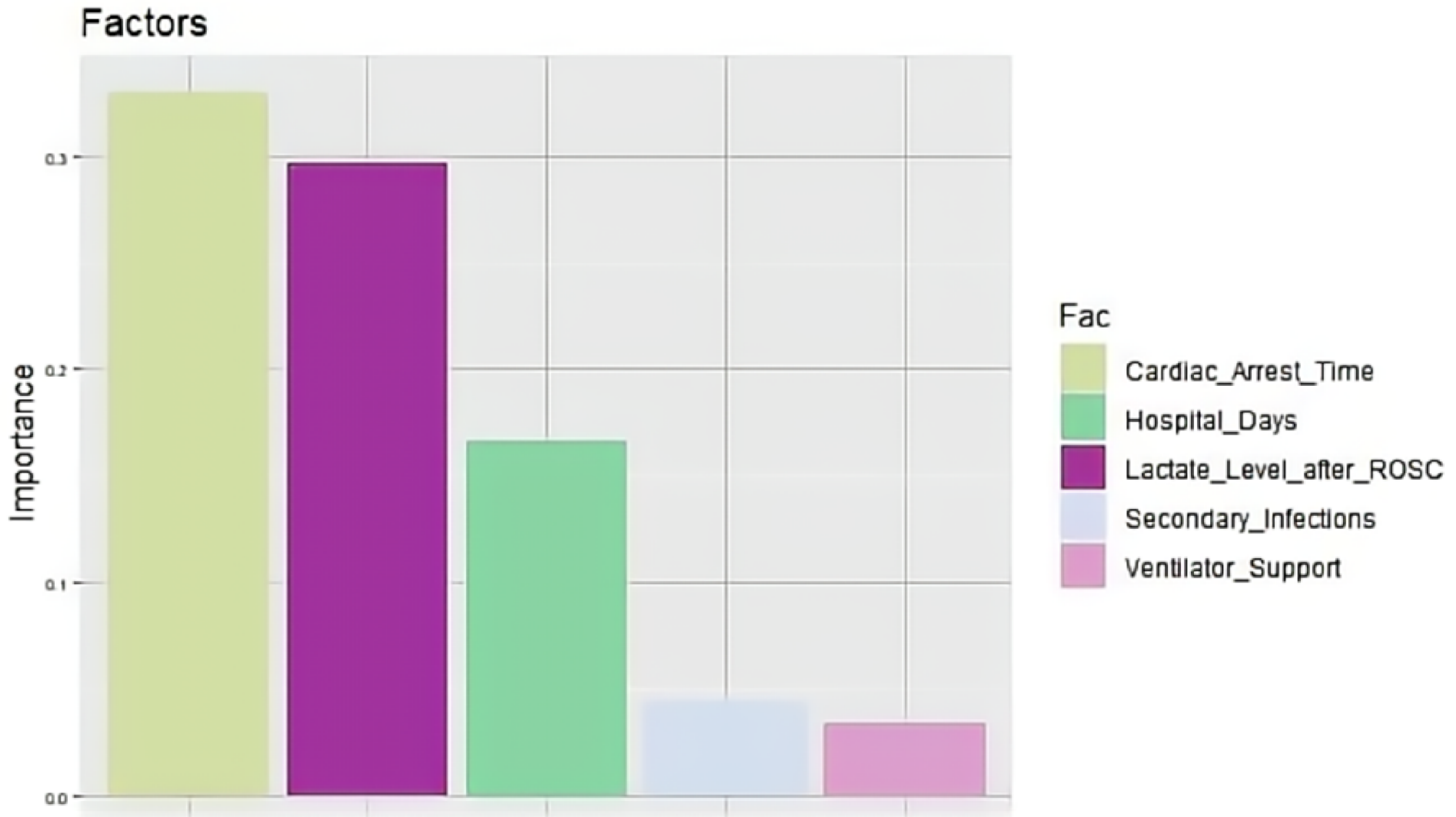
Importance ranking of all risk factors.

A total of 221 patients who experienced CA and ROSC were admitted to Fujian Medical University Union Hospital between January 2018 and June 2022. We excluded 22 patients for the following reasons: 8 refused invasive rescue by relatives, 9 had missing key data, and 5 were lost to follow-up. Of the remaining 199 patients, 95 were still alive and 104 had died within 6 months after CA. The comparisons of baseline characteristics between alive and dead groups are presented in Table 1. Duration of cardiac arrest, lactate level after ROSC, secondary infections, length of hospital stay, and ventilator support were the top five risk factors of death within 6 months after CA in sequence (*P* < 0.1) (Table 2 and Figure 1).

**Table 1:** Baseline characteristics of the alive group and the dead group.

Characteristics	Alive group (*n* = 95)	Dead group (*n* = 104)	*P-*value
Age (mean ± SD), years	58.05 ± 16.39	61.13 ± 14.96	0.168
≥60 years, No. (%)	46 (48.42%)	55 (52.88%)	0.529
Sex, No. (%)			0.295
Male	61 (64.21%)	74 (71.15%)	
Female	34 (35.79%)	30 (28.85%)	
Length of hospital stay (mean ± SD), days	25.19 ± 37.57	14.43 ± 22.78	**0**.**015**
Fee (mean ± SD), yuan	126,372.73 ± 150,194.47	105,908.85 ± 196,023.99	0.413
Duration of cardiac arrest (mean ± SD), min	11.18 ± 9.97	34.84 ± 30.37	**0**.**000**
Cardiac arrest location, No. (%)			0.138
In-hospital	78 (82.11%)	93 (88.57%)	
Out-of-hospital	17 (17.89%)	78 (74.29%)	
Ventilator support, No. (%)	64 (67.37%)	87 (83.65%)	**0**.**007**
Time on the ventilator (mean ± SD), h	197.28 ± 276.81	172.91 ± 276.81	0.606
GCS score on admission (mean ± SD) score	9.74 ± 4.84	5.52 ± 4.62	**0**.**000**
GCS score at discharge (mean ± SD), score	11.74 ± 4.84	3.49 ± 2.33	**0**.**000**
Lactate level after ROSC (mean ± SD), mmol/L	3.97 ± 3.84	9.76 ± 5.51	**0**.**000**
Tracheostomy, No. (%)	18 (18.95%)	6 (5.77%)	**0**.**004**
Causes of cardiac arrest, No. (%)			**0**.**00745**
Cardiovascular	57 (60.00%)	49 (47.12%)	
Neurogenic	4 (4.21%)	10 (9.62%)	
Respiratory	14 (14.74%)	21 (20.19%)	
Infection	2 (2.11%)	13 (12.50%)	
Other	18 (18.95%)	11 (10.58%)	
Hypothermia, No. (%)	4 (4.21%)	2 (1.92%)	0.346
Subsequent epilepsy, No. (%)	11 (11.58%)	10 (9.62%)	0.652
Secondary infections, No. (%)	58 (61.05%)	84 (80.77%)	**0**.**002**

GCS, Glasgow coma scale; SD, standard deviation; No., number; ROSC, return of spontaneous circulation.

A chi-square test was used to compare categorical variables, and the Wilcoxon two-sample test was performed to compare continuous variables.

Bold values mean statistical significance.

**Table 2:** Risk factors for death within 6 months after CA.

Factors	Odds	95% CI	*P*-value
Hospital days	0.978	0.960–0.997	**0**.**020**
Cardiac arrest reason	1.202	0.915–1.578	0.186
Duration of cardiac arrest	1.065	1.032–1.098	**0**.**000**
Ventilator support	0.323	0.104–0.997	**0**.**049**
Tracheostomy	0.136	2.545–0.745	0.136
Lactate level after ROSC	1.229	1.122–1.345	**0**.**000**
Secondary infections	0.206	0.071–0.599	**0**.**004**

ROSC, return of spontaneous circulation; CI, confidence interval.

Bold values mean statistical significance.

### Model development and external validation

**Figure 2:**
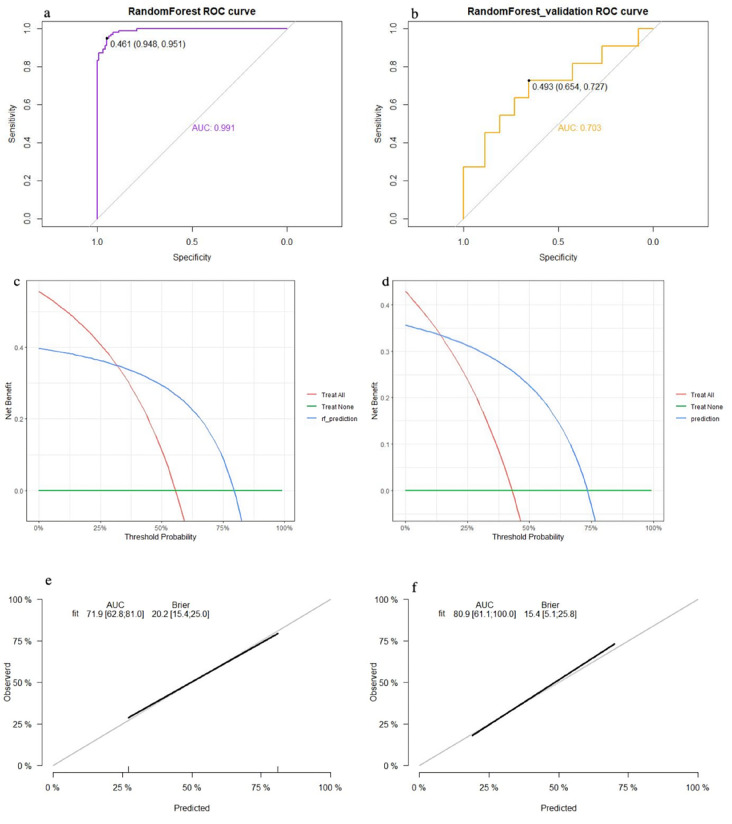
Performance of the random forest model: (**a**) receiver operating characteristic curve of the training set; (**b**) receiver operating characteristic curve of the validation set; (**c**) decision curve analysis of the training set; (**d**) decision curve analysis of the validation set; (**e**) calibration curve of the training set; and (**f**) calibration curve of the validation set.

The top five factors with *P* < 0.1 in the multivariate analysis were used to establish predictive models using random forest, support vector machine (SVM), and nomogram methods, with external validation conducted for each. In the training set, the random forest model achieved an AUC of 0.991, an accuracy of 81.0%, a sensitivity of 79.0%, and a specificity of 84.4% (Figure 2a). In the validation set, the random forest model also showed good discrimination, with an AUC of 0.703, an accuracy of 68.5%, a sensitivity of 85.7%, and a specificity of 47.2% (Figure 2b). The decision curve analysis and calibration curve results for the training set (Figures 2c,e) and the validation set (Figures 2d,f) are illustrated in Figure 2.

**Figure 3:**
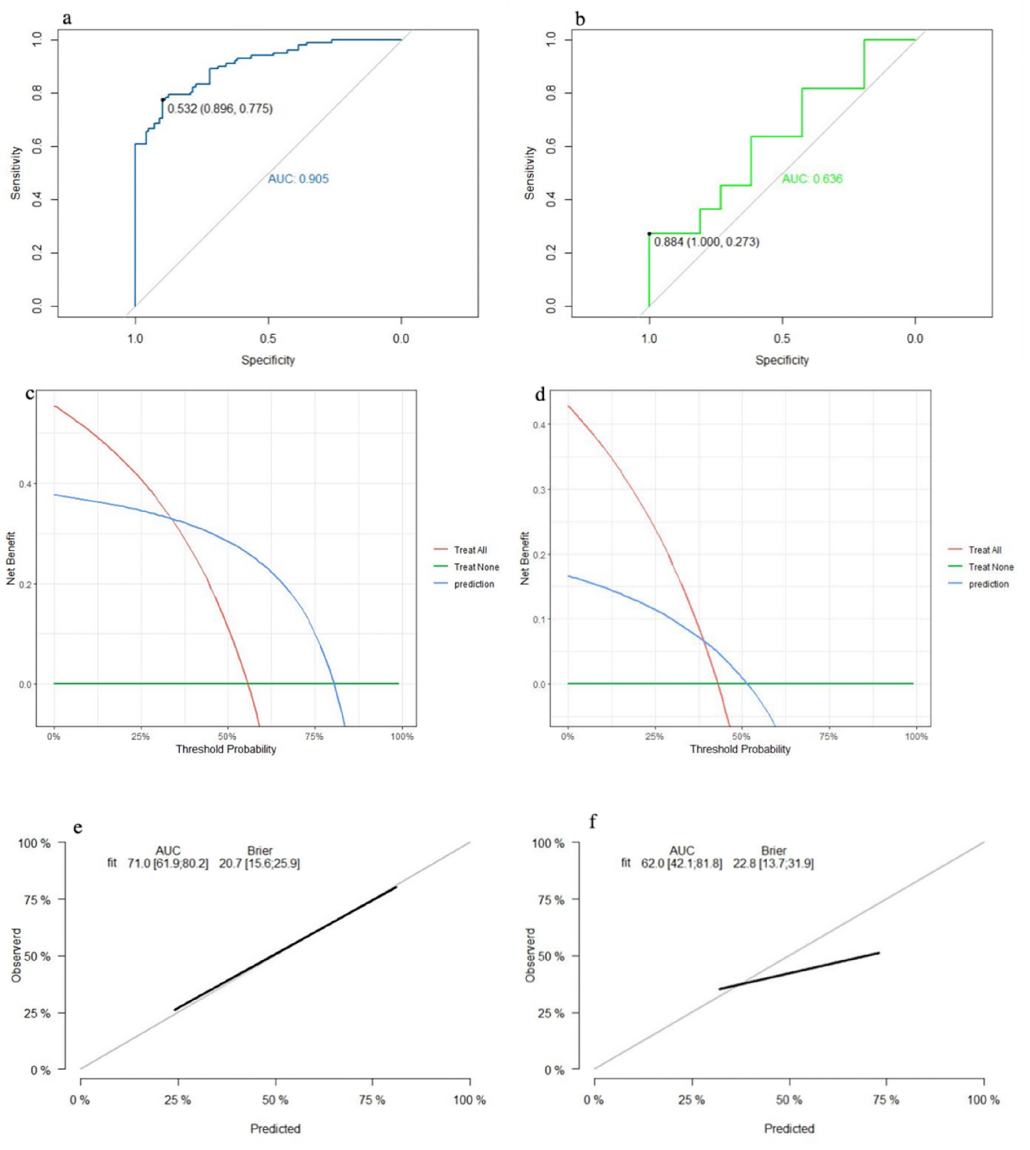
Performance of the SVM model: (**a**) receiver operating characteristic curve of the training set; (**b**) receiver operating characteristic curve of the validation set; (**c**) decision curve analysis of the training set; (**d**) decision curve analysis of the validation set; (**e**) calibration curve of the training set; and (**f**) calibration curve of the validation set.

**Figure 4:**
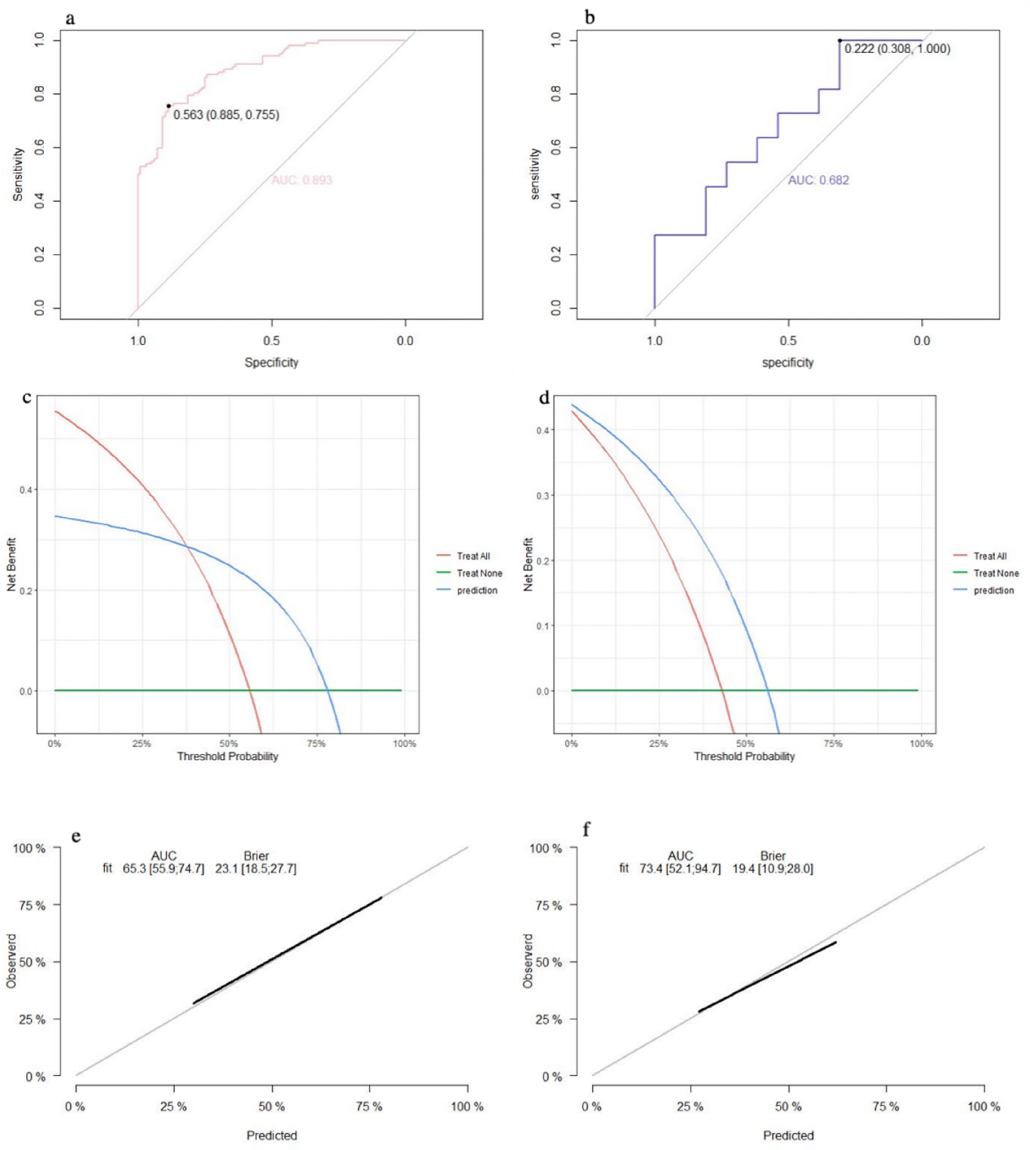
Performance of the nomogram model: (**a**) receiver operating characteristic curve of the training set; (**b**) receiver operating characteristic curve of the validation set; (**c**) decision curve analysis of the training set; (**d**) decision curve analysis of the validation set; (**e**) calibration curve of the training set; and (**f**) calibration curve of the validation set.

**Figure 5:**
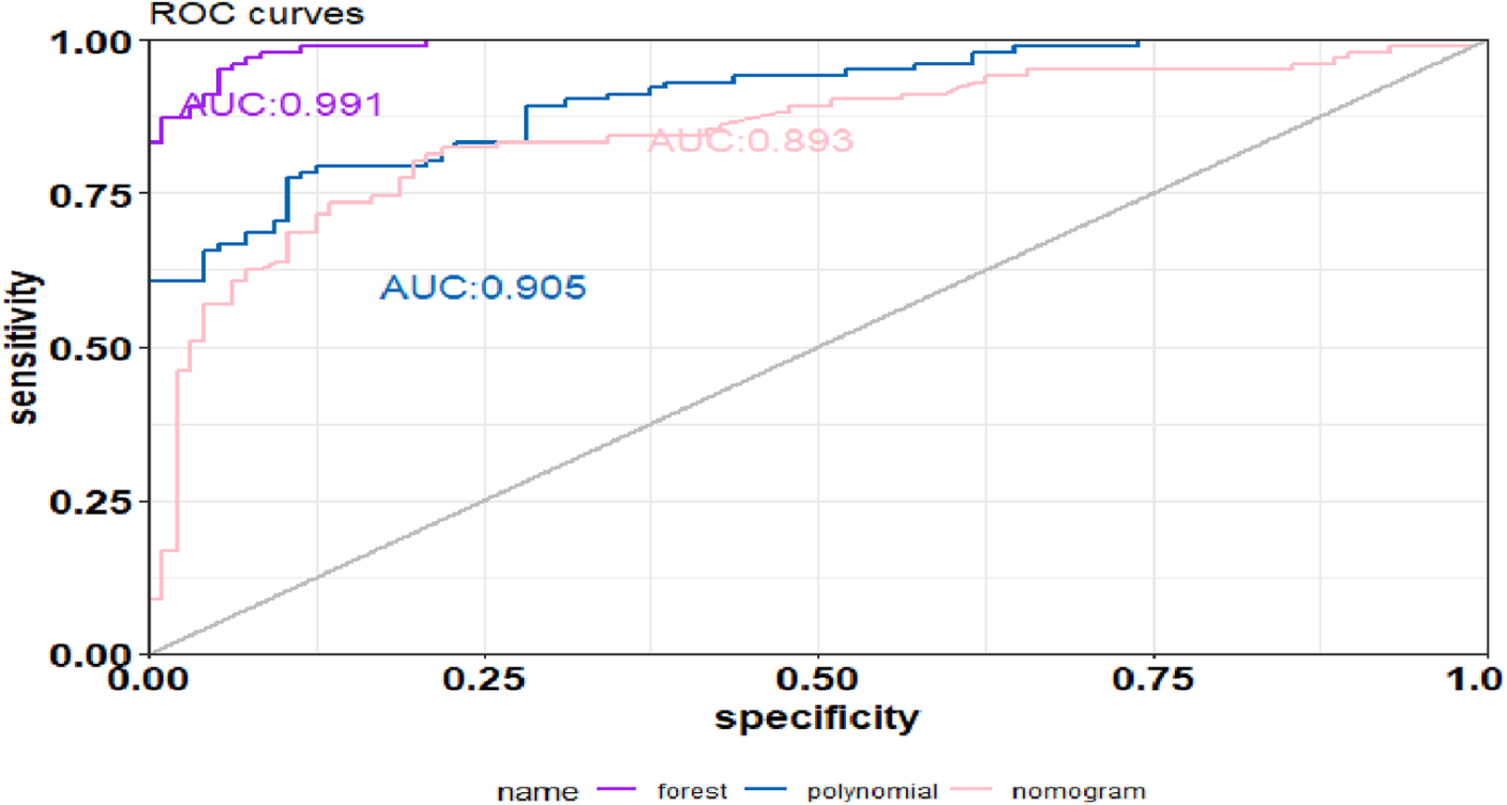
Comparison of AUC–ROC curves across all models.

The SVM model showed a mean accuracy of 80.2% and a mean AUC of 0.905 in the training set and a mean accuracy of 77.0% and a mean AUC of 0.636 in the validation set (Figure 3). The nomogram model demonstrated an accuracy of 80.7% and an AUC of 0.893 in the training set and an accuracy of 70.7% and an AUC of 0.632 in the validation set (Figure 4). The average AUC of the prediction model constructed using random forest, SVM, and nomogram methods was 0.991, 0.893, and 0.905, respectively (Figure 5).

## Discussion

In this study, we demonstrated that the top five risk factors for death within 6 months after CA, in sequence, were duration of cardiac arrest, lactate level after ROSC, secondary infections, length of hospital stay, and ventilator support. The predictive models constructed using random forest, SVM, and nomogram methods based on these above factors performed well in the training set. Among the three models, the random forest model exhibited the best prediction performance in the external validation set.

CA is a major cause of death and a highly disabling condition worldwide (22–24). Even in patients who obtain ROSC, the prognosis remains very poor. The prognosis of CA patients is mainly determined by the extent of neurological injury induced by circulatory arrest. Various factors and biomarkers have been studied as early prognostic markers for CA patients who achieve ROSC (25–27). The duration of CA is crucial for patient outcomes. In some CA models, neuronal injury highly depends on the duration of CA, with longer durations consistently associated with a large increase in mortality (28, 29). Among the laboratory parameters obtainable in the early hours after ROSC, lactate levels have been used to predict outcomes after CA. Particularly, lactate level on admission has the diagnostic ability to predict neurological outcomes after CA (30). In this study, lactate levels were measured in the first 24 h after ROSC. Poor outcomes were observed in CA patients who required ventilator support. The results of risk factors for the prognosis of CA in this study are largely consistent with findings from previous reports.

Various studies have proposed different approaches to predict neurological and other clinical outcomes for CA patients (31–33). For example, scoring models have been to predict the neurological outcomes for CA patients (34–36). Differences exist among different studies: (1) data sets vary, with some being larger but more heterogeneous, leading to variations in model performance due to differences in characteristics, care practices, and clinical care; (2) model training and validation strategies differ across studies, with some studies even having not performed validation; (3) model evaluation practices differ across studies; (4) most studies have constructed only one model. In this study, more than 200 patients were enrolled to develop different predictive models, and prospective validation was conducted. In addition, we used model calibration and decision analysis, in addition to AUC, for evaluation. Calibration measures a model's ability to provide clinically relevant probabilistic estimates of risk, which can be done at the individual patient level and across all predicted probabilities. Decision analysis is an objective, explicit method that uses models to represent specific decision problems. Like most studies, predictive models tend to perform more poorly on the external validation set than on the training and test sets.

Overall, the contributions of the present work include technical innovations such as complete use of the data, effective utilization of temporal information, and increased automation; increased rigor in investigating model generalizability; and enhanced rigor and scale of model validation. One limitation of these prediction models is that they could only predict survival or death. It would be more meaningful to assess neurological outcomes beyond the Glasgow coma scale (GCS) at discharge, especially after 6 months. Since this was a retrospective study, outcome follow-ups were conducted by telephone, and further assessment could not be performed.

## Conclusions

In conclusion, this study developed and validated a prediction model using early post-arrest factors, which can provide suggestions for evaluating prognosis within 6 months after cardiac arrest. The predictive model constructed using the random forest method demonstrated better predictive efficacy. Nevertheless, our conclusions still need to be further verified by well-designed, prospective cohort studies, and additional data are needed to validate these models.

## Data Availability

The original contributions presented in the study are included in the article/Supplementary Material; further inquiries can be directed to the corresponding author.
